# Messenger RNA of *FCGR3A* and *FCGR3B* Genes as Monitoring Markers of Clear Cell Renal Adenocarcinoma (a Pilot Study)

**DOI:** 10.17691/stm2022.14.3.03

**Published:** 2022-05-28

**Authors:** A.V. Alyasova, Z.V. Amoev, O.O. Shkola, D.V. Novikov, S.G. Selivanova, V.V. Novikov

**Affiliations:** Professor, Department of Oncology, Radiation Therapy and Radiation Diagnostics; Privolzhsky Research Medical University, 10/1 Minin and Pozharsky Square, Nizhny Novgorod, 603005, Russia; Urologist; Volga District Medical Centre of Federal Medical Biological Agency of Russia, 14 Ilyinskaya St., Nizhny Novgorod, 603109, Russia; PhD Student, Department of Molecular Biology and Immunology; National Research Lobachevsky State University of Nizhni Novgorod, 23 Prospekt Gagarina, Nizhny Novgorod, 603950, Russia; Leading Researcher; Blokhina Scientific Research Institute of Epidemiology and Microbiology of Nizhny Novgorod, Federal Service for Surveillance on Consumer Rights Protection and Human Wellbeing (Rospotrebnadzor), 71 Malaya Yamskaya St., Nizhny Novgorod, 603950, Russia; Senior Researcher; Blokhina Scientific Research Institute of Epidemiology and Microbiology of Nizhny Novgorod, Federal Service for Surveillance on Consumer Rights Protection and Human Wellbeing (Rospotrebnadzor), 71 Malaya Yamskaya St., Nizhny Novgorod, 603950, Russia; Professor, Department of Molecular Biology and Immunology; National Research Lobachevsky State University of Nizhni Novgorod, 23 Prospekt Gagarina, Nizhny Novgorod, 603950, Russia; Leading Researcher; Blokhina Scientific Research Institute of Epidemiology and Microbiology of Nizhny Novgorod, Federal Service for Surveillance on Consumer Rights Protection and Human Wellbeing (Rospotrebnadzor), 71 Malaya Yamskaya St., Nizhny Novgorod, 603950, Russia Head of the Laboratory of Immunochemistry; Blokhina Scientific Research Institute of Epidemiology and Microbiology of Nizhny Novgorod, Federal Service for Surveillance on Consumer Rights Protection and Human Wellbeing (Rospotrebnadzor), 71 Malaya Yamskaya St., Nizhny Novgorod, 603950, Russia

**Keywords:** monitoring renal cancer markers, mRNA of *FCGR3A* gene, mRNA of *FCGR3B* gene

## Abstract

**The aim of the study** was to assess the capabilities of mRNA genes encoding CD16a (*FCGR3A*) and CD16b (*FCGR3B*) in tumor samples from patients with renal cancer, and characterize the tumor process in relation to clinical and morphological factors.

**Materials and Methods:**

We used 125 tumor samples from patients with a histologically confirmed diagnosis of renal cancer T_1–4_N_0–1_M_0–1_. A method described by Chomczynski and Sacchi was used to isolate nucleic acids. The mRNA levels were determined using a reverse transcription polymerase chain reaction and calculated according to ΔΔCt formula, taking into account the reaction efficiency.

**Results:**

mRNA of the *FCGR3A* gene was detected in all tumor tissue samples under study; in contrast, mRNA of the *FCGR3B* gene was found only in 92.0% (115/125) of cases. In tumors classified as pT1, the mRNA content of the *FCGR3A* gene was significantly lower than that in tumor samples of pT_3_ size. There was the significant increase in the mRNA content of both genes with an increase in tumor grade, as well as in the cases with distant metastases. The presence of a tumor thrombus in the inferior vena cava system was accompanied by a significant increase in the mRNA content of the *FCGR3A* gene.

**Conclusion:**

In tumor tissue samples from patients with clear cell renal cancer, the predominant production of the *FCGR3A* mRNA was observed in comparison with the *FCGR3B* mRNA. The revealed relationship of an increased amount of the *FCGR3A* mRNA and, in some cases, the *FCGR3B* mRNA with a number of clinical and morphological factors enables to consider the mRNA level of the genes as new monitoring biomarkers.

## Introduction

Renal cell carcinoma amounts to 80–85% from renal cancers, and 2–3% — from all carcinomas [1]. Despite the progress in diagnostics, its surgical and medical therapy, a clinical outcome is still not satisfactory [2]. One of the reasons is the lack of biomarkers enabling to control the disease course and individualize the treatment [3].

The immune system of the body plays a prominent role in the tumor growth process; it controls the tumor development and metastases spreading [4]. An immune response involves natural killers (NK cells), neutrophils, monocytes/macrophages. For monitoring purposes, the expression assessment of *FCGR3A* and *FCGR3B* genes encoding CD16a and CD16b proteins can be used. CD16a expression is typical for natural killers. In addition, CD16a is found on the membrane of monocytes, tissue-specific macrophages, γδ T lymphocytes and dendritic cells [5, 6]. CD16b protein is a molecular marker of neutrophils [7]. Moreover, CD16b at a lower level expresses on basophils and is revealed on eosinophils after IFN-_γ_ induction [2].

The assessment of CD16 membrane molecule expression is used to determine the population structure of peripheral blood cells and their functional state in oncological conditions. There is rather limited information on the interaction between the levels of mRNA genes encoding CD16a (*FCGR3A*) and CD16b (*FCGR3B*) in a tumor, and clinic and morphological factors determining a cancerous disease course including renal cancer. However, no detailed studies of *FCGR3A* and *FCGR3B* have been carried out at a transcription level.

**The aim of the study** was to assess the capabilities of mRNA genes encoding CD16a (*FCGR3A*) and CD16b (*FCGR3B*) proteins in tumor samples from patients with renal cancer, and characterize the tumor process in relation to clinical and morphological factors.

## Materials and Methods

In the present study, we used 125 tumor samples from patients with a histologically confirmed diagnosis of renal cancer T_1–4_N_0–1_M_0–1_. The tumor tissue samples were obtained during operational interventions — radical or partial nephrectomy. Among the patients under study, there were 63.2% men (79/125) and 36.8% women (46/125); the mean age of patients was 60.2 years. 44.0% of study subjects (55/125) had T_1_ tumor grade; 6.4% (8/125) — T_2_; 49.6% (62/125) — T_3_. The tumor grade according to Fuhrman was as follows: GI — 16.8% (21/125) of patients; GII — 17.6% (22/125); GIII — 57.6% (72/125); GIV — 8% (10/125). Lymph node lesions were revealed in 17.6% of cases (22/125), the presence of distant metastases was found in 40.3% (31/125), namely: in adrenal glands — in 22.6% of cases (7/31); in the lungs — in 29.0% (9/31); in bones — in 25.8% (8/31); in the liver — in 22.6% (7/31). A tumor thrombus in the inferior vena cava system was found in 25.6% cases (32/125).

The diagnostic and treatment extent of renal cancer patients were in accordance with the recommended diagnostics and treatment algorithms for malignancies approved by the Ministry of Health of the Russian Federation. The present study was carried out according to the declaration of Helsinki (2013) and was approved by the Ethics Committee of Volga District Medical Centre of Federal Medical Biological Agency of Russia (Nizhny Novgorod, Russia). Each patient gave an informed consent.

A method described by Chomczynski and Sacchi [8] was used to isolate nucleic acids. Complementary DNA was synthesized using an M-MLV reverse transcriptase (Sileks, Russia) according to the manufacturer’s recommendations. The relative level of *FCGR3A* and *FCGR3B* mRNA was determined by a real-time polymerase chain reaction (PCR) using CFX96 Touch amplifiers (Bio-Rad, USA) within 1 h 15 min according to the following program: 94.0°С — 2 min (42 20-second PCR cycles); 60.0°С — 20 s; 72.0°С — 20 s. The reaction mixture contained: 4.1 μl of Н_2_О (bidistilled water); 1.5 μl of 10х buffer; 1.2 μl of MgCl_2_; 1.2 μl of deoxynucleoside triphosphates (dNTP); 0.2 μl of HotTaq polymerase (Sileks), and 1.8 μl of each pligonucleotide (Syntol, Russia), their sequence is presented in Table 1.

**Table 1:** Sequence of oligonucleotides used for reactions

Oligonucleotide structure		
Gene	Oligonucleotide	Primary structure (5’–3’)
*FCGR3A*	Uni-F	CAGCTGGCATGCGGACTGA
	RA-R	CACTGTCCTTCTCGAGCACC
	FAB Z	ROX-CTGTGGTGTTCCTGGAGCCTCAATGGTA-BHQ-2
*FCGR3B*	Uni-F	CAGCTGGCATGCGGACTGA
	RB-R	CACTGTCCTTCTCAAGCACG
	FAB Z	ROX-CTGTGGTGTTCCTGGAGCCTCAATGGTA-BHQ-2
Ubiquitin C	UBC F	GCACAGCTAGTTCCGTCGCA
	UBC R	TGCATTGTCAAGTGACGAT
	UBC Z	CY5-ATTTGGGTCGCAGTTCTTGTTTGTGGAT-BHQ-2

Levels of mRNA were counted according to ΔΔCt formula, considering the reaction efficiency [9]. They were normalized according to a relative level of mRNA ubiquitin С (UBC).

### Statistical data processing

The findings were statistically processed using Statistica v. 8.0. Shapiro– Wilk test was used to test a hypothesis on the compliance of a relevant distribution of the obtained variants with normal distribution. Median (Ме), quartiles Q1 (25%) and Q3 (75%) were counted to determine quantitative indices. Mann–Whitney two-tailed test was applied to compare two independent groups by quantitative properties, and Kruskal–Wallis test was used to compare three and more independent groups. The differences between the groups were considered statistically significant if p<0.05.

## Results and Discussion

The conducted researches showed mRNA of the *FCGR3A* gene to be revealed in all tumor tissue samples. In contrast, mRNA of the *FCGR3В* gene was found only in 92.0% cases (115/125). The *FCGR3A* mRNA content in a tumor was 2.2 times as high (p<0.05) as *FCGR3В* (Table 2).

**Table 2:** Content of mRNA of *FCGR3A* and *FCGR3В* genes in renal cancer patients, Me [25%; 75%]

Groups	mRNA *FCGR3A*	mRNA *FCGR3В*
Men (n=79)	0.269 [0.147; 0.413]	0.102 [0.021; 0.187]
Women (n=46)	0.219 [0.137; 0.314]^#^	0.128 [0.037; 0.233]
Total (n=125)	0.238 [0.144; 0.379]	0.109 [0.026; 0.208]*

Notes. The differences are significant (p<0.05): * when compare the mRNA content of *FCGR3A* and *FCGR3B*; ^#^ when compare the mRNA content of *FCGR3A* in male and female patients.

Thus, renal cancer development was accompanied by the predominant activation of *FCGR3A* gene expression that can be the evidence of a high tumor infiltration degree, primarily, NK cells.

The mRNA level of *FCGR3A* gene in male tumor samples was higher than in female ones by 1.2 times (p>0.05) (see Table 2). Gender differences of immunological indices are given in literature, for instance, in colorectal cancer patients [10, 11]. The mRNA content of *FCGR3В* gene in both groups had no significant differences.

In patients with tumors classified as рТ_1_, the mRNA content of the *FCGR3А* gene was significantly lower (p<0.05) and in the samples of patients with рТ_3_ tumors — by 1.5 times as high (Table 3). No differences were found in the *FCGR3В* mRNA levels in the patients with a primary lesion of various sizes (р>0.05). It should be noted that there were significant differences in the amounts of mRNA of these genes in tumor samples classified as рТ_2_, where the *FCGR3А* mRNA level 10 times exceeded the *FCGR3В* mRNA content (р<0.05). In рТ_1_ and рТ_3_ tumor samples, the content of the tested mRNA of the genes *FCGR3А* and *FCGR3В* had no differences (р>0.05), while the *FCGR3А* mRNA level was higher than the *FCGR3В* mRNA content only by 2.2 and 2.0 times, respectively.

**Table 3:** Content of mRNA of *FCGR3A* and *FCGR3В* genes in renal cancer patients depending on a primary lesion size and tumor grade, Me [25%; 75%]

Groups	mRNA *FCGR3A*	mRNA *FCGR3В*
Size:		
рТ_1_ (n=55) (1)	0.199 [0.103; 0.298]	0.091 [0.026; 0.175]
рТ_2_ (n=8) (2)	0.462 [0.241; 0.503]	0.046 [0.010; 0.081]*
рТ_3_ (n=62) (3)	0.305 [0.193; 0.450]	0.147 [0.034; 0.406]
	p_1–3_<0.05	
Tumor grade:		
GI (n=21) (4)	0.147 [0.082; 0.229]	0.099 [0.063; 0.166]
GII (n=22) (5)	0.227 [0.144; 0.316]	0.076 [0.020; 0.178]
	p_4–5_<0.05	
GIII (n=72) (6)	0.379 [0.239; 0.530]	0.126 [0.002; 0.598]
	p_4–6_<0.05	
GIV (n=10) (7)	0.439 [0.404; 0.763]	0.435 [0.207; 0.737]
	p_4–7_<0.05	p_4–7_<0.05

^*^ the differences are significant when compare the *FCGR3A* and *FCGR3В* mRNA content inside the рТ_2_ group (p<0.05).

It should be mentioned that the nature of the change in mRNA levels of the genes in these groups is differently directed (see Table 3). The *FCGR3А* mRNA content is likely to be the more sensitive marker of the primary tumor growth than the *FCGR3В* mRNA amount. In literature, there is the information on possible involvement of *FCGR3A* in controlling chronic inflammation in the tumors in colorectal cancer patients [10]. It is entirely possible that there are similar mechanisms of *FCGR3A* involvement in renal cancer pathogenesis.

The study assessed the content of *FCGR3А* and *FCGR3В* mRNA in tumors of different grades (GЗ). In GII, the *FCGR3А* mRNA levels increased by 1.5 times (р<0.05) compared to its value in the samples of tumor GI patients, in GIII — by 2.6 times (р<0.05), in GIV — by 2.9 times (р<0.05), respectively (see Table 3). The *FCGR3В* mRNA amount in GIV tumor samples increased by 4.4 times (р<0.05) compared to its amount in tumor GI samples.

The findings showed the changes in mRNA under study to be one-directional related to the growth of both the *FCGR3А* mRNA content and the *FCGR3В* mRNA content if the tumor grade increased. The revealed differences of mRNA content of these genes in patients with tumors of different grades showed that if tumor grade increases, the immune response intensity grows that is not optimal for tumor growth inhibition. Presumably, it is related to the involvement of a tumor itself into the expression processes of membrane proteins on the surface of immunocompetent cells [12]. Moreover, there is a strong possibility that when a primary tumor grows or its grade increases, the intensity of inflammatory processes in the surrounding tissues increases as well and it is accompanied by attracting to the tumor different leukocytic cells including neutrophils, NK cells, and macrophages.

The presence or absence of metastases in regional lymph nodes resulted in no significant differences in the mRNA amount of *FCGR3А* and *FCGR3В* genes in patients’ tumor tissue samples (p>0.05). And in contrast, if there were distant metastases, *FCGR3А* mRNA level was higher by 1.7 times (p<0.05), and *FCGR3В* mRNA content was by 1.2 as high (p<0.05) compared to the patients who had no secondary lesions (Table 4).

**Table 4:** *FCGR3A* and *FCGR3В* mRNA content in renal cancer patients depending on the presence of distant metastases and tumor thrombi, Me [25%; 75%]

Groups	mRNA *FCGR3A*	mRNA *FCGR3В*
No metastases (n=94) (1)	0.226 [0.138; 0.330]	0.102 [0.033; 0.190]
	p_1–2_<0.05	p_1–2_<0.05
Presence of metastases (n=31) (2)	0.376 [0.185; 0.433]	0.126 [0.015; 0.219]
Presence of thrombus (n=32) (3)	0.381 [0.217; 0.485]	0.127 [0.036; 0.305]
No thrombus (n=93) (4)	0.221 [0.135; 0.314]	0.102 [0.024; 0.188]
	p_3–4_<0.05	

In case of a tumor thrombus in the inferior vena cava system, *FCGR3А* mRNA content increased by 1.7 times compared to those patients who had no thrombus revealed (p<0.05). The amount of *FCGR3В* mRNA had no significant difference in both groups.

Distant metastases and/or tumor thrombi revealed in the inferior vena cava system refer to unfavorable prognostic factors [13-15]. One might assume that increased infiltration of a tumor by neutrophils and NK cells is observed in more unfavorable cases. Natural killers play a key role in protecting against malignant or virus-infected cells [16]. They can be “specifically activated” through certain Fc receptors which express on their cell surface including Fc_γ_RIIIa transmitting activating signals inside a cell. After Fc receptors are activated by antibodies related to targeting cells, NK cells enable to lyse targeting cells without priming and secrete cytokines such as gamma interferon [17, 18]. High level of *FCGR3А* mRNA under these conditions is likely to be related to an increased expression of Fc_γ_RIIIa aimed at the activation of NK cells, and therefore, in some particularly unfavorable situations there is an increase in *FCGR3В* mRNA level.

The relationship between renal cell carcinoma infiltration by immune cells and clinic and pathological characteristics are still unclear and under extensive study. The composition of tumor infiltrating leukocytes is rather heterogeneous. The leukocytes infiltrating a tumor include lymphocytes (CD8_+_ Т cells, Th1 cells, В lymphocytes), dendritic cells, tumor-associated macrophages, neutrophils, NK cells. Th2 and suppressor cells are found relatively rarely, suggesting the predominant anti-inflammatory profile. Relatively recently, the T lymphocyte population was shown by mass-spectrometry to prevail; they are followed by tumor-associated macrophages performing Fc_γ_R-mediated phagocytosis, NK cells, В cells, dendritic cells, and neutrophils [19]. Along with suppressor cells, the tumor microenvironment includes suppressor myeloid cells infiltrating a tumor, blocking the development of an effective immune response [20].

Another type of immune cells contributing to tumor suppression is tumor-associated macrophages [21]. No response to tyrosine kinase inhibitors was found to be related to an increased number of activated tumor-associated macrophages in tumor lesions [19]. Tumor-associated NK cells were revealed to have low CD16 expression, although their number in percentage terms is eightfold as much as the content of the same cells in blood. In addition, in different patient the variability of the number of NK cells is high, depending on a tumor process and having an effect on tumor microenvironment [22]. Infiltration degree by NK cells and the expression of markers (CD16 and cytokines) was shown to determine the functional capacity of NK cells infiltrating renal cell carcinoma, and can be used to characterize the renal cell carcinoma subgroups [23]. Positive correlations between the content of macrophages, a tumor stage, and a tumor grade have been found. Moreover, tumor-associated neutrophils were found to contribute to tumor developing through antitumor immunity restrictions by affecting local inflammation, angiogenesis, and lymphangiogenesis [24]. Our findings suggest an increased expression level of the genes encoding CD16a in NK cells, macrophages, CD16b — in neutrophils, and are consistent with the information reported in literature.

## Conclusion

In tumor tissue samples from patients with clear cell renal cancer, the predominant production of the *FCGR3A* mRNA was observed in comparison with the *FCGR3B* mRNA. The content of *FCGR3А* mRNA increases in case there are clinical and morphological characteristics of an unfavorable prognosis: an increasing tumor grade, distant metastases, a tumor thrombus in the inferior vena cava system.

The *FCGR3А* mRNA content in tumor tissue of the patients is more exposed to quantitative changes related to the revealed unfavorable prognostic factors than the *FCGR3В* mRNA level. However, in the cases of low-graded tumors or secondary lesions, the production of *FCGR3В* mRNA increases compared to its values in more prognostically favorable conditions.

The mRNA expression level of *FCGR3А*, and in some cases — the *FCGR3В* mRNA in tumor tissue in renal cancer patients enables to suggest a prognostic value of the genes to assess the disease course.
